# HDAC Activity Is Required during *Xenopus* Tail Regeneration

**DOI:** 10.1371/journal.pone.0026382

**Published:** 2011-10-14

**Authors:** Ai-Sun Tseng, Kátia Carneiro, Joan M. Lemire, Michael Levin

**Affiliations:** 1 Department of Developmental Biology, Center for Regenerative and Developmental Biology, Tufts University, Medford, Massachusetts, United States of America; 2 Department of Histology and Embryology, Institute of Biomedical Sciences, Federal University of Rio de Janeiro, Rio de Janeiro, Brazil; Oxford Brookes University, United Kingdom

## Abstract

The ability to fully restore damaged or lost organs is present in only a subset of animals. The *Xenopus* tadpole tail is a complex appendage, containing epidermis, muscle, nerves, spinal cord, and vasculature, which regenerates after amputation. Understanding the mechanisms of tail regeneration may lead to new insights to promote biomedical regeneration in non-regenerative tissues. Although chromatin remodeling is known to be critical for stem cell pluripotency, its role in complex organ regeneration *in vivo* remains largely uncharacterized. Here we show that histone deacetylase (HDAC) activity is required for the early stages of tail regeneration. HDAC1 is expressed during the 1^st^ two days of regeneration. Pharmacological blockade of HDACs using Trichostatin A (TSA) increased histone acetylation levels in the amputated tail. Furthermore, treatment with TSA or another HDAC inhibitor, valproic acid, specifically inhibited regeneration. Over-expression of wild-type Mad3, a transcriptional repressor known to associate in a complex with HDACs via Sin3, inhibited regeneration. Similarly, expression of a Mad3 mutant lacking the Sin3-interacting domain that is required for HDAC binding also blocks regeneration, suggesting that HDAC and Mad3 may act together to regulate regeneration. Inhibition of HDAC function resulted in aberrant expression of Notch1 and BMP2, two genes known to be required for tail regeneration. Our results identify a novel early role for HDAC in appendage regeneration and suggest that modulation of histone acetylation is important in regenerative repair of complex appendages.

## Introduction

Tadpoles of the African clawed frog, *Xenopus laevis*, have the ability to rapidly regenerate their tails upon amputation [1], [2], [3], [4]. The tail is a complex, highly-patterned appendage consisting of multiple tissues including epidermis, muscle, spinal cord, nerves and vasculature. Thus an understanding of how natural regeneration occurs may provide approaches for developing human regenerative repairs.

Recent studies have identified a set of processes that occur when the tail is lost. Amputation of a tail results in migration of the epidermal cells to cover the wound within 2–3 hours [5]. By 24 hours post amputation (hpa), a swelling called the regeneration bud is formed at the wound site. This regeneration bud contains the progenitor cells necessary to re-grow the entire appendage properly. Notably, grafting experiments have shown that these progenitors are lineage-restricted and will only reconstitute their particular tissue type; no metaplasia has been observed. Within 2 weeks, an entirely new tail is fully regenerated [3], [4] via a process that comprises both bioelectrical [6], [7], [8] and biochemical [5], [9], [10], [11], [12] signaling pathways.

The critical process of tail regeneration requires that cells re-enter the cell cycle and differentiate from a lineage-restricted progenitor cell population into a specific cell type to replace the damaged tissue and reconstitute the missing tissue. Organ rebuilding using the newly generated cells must also be orchestrated in three dimensions in order to properly restore the complex morphology of the intact neuromuscular appendage. Thus, tail regeneration is a tractable *in vivo* model well-suited to understand how differentiated cell types can transiently convert to a highly proliferative state that also recapitulates developmental gene expression programs [13], [14]. How do the cells involved in tail regeneration revert to a highly proliferative state? How this state is achieved and executed at the molecular levels is of great interest because of its relevance to regenerative strategies for human tissue and/or organ repair.

The proliferation of differentiated somatic cells upon injury is a process mostly dictated by the epigenetic markers they harbor on regulatory regions of tissue specific genes [15]. In contrast to epigenetic modifications that occur on a genome-wide scale during the initial stages of animal development, the epigenome of somatic cells is generally stable. Thus, in order to re-enter the cell cycle, somatic cells must undergo remodeling of the epigenetic landscape from its differentiated epigenetic program to a highly proliferative state through chromatin remodeling [16], [17].

One important aspect of chromatin remodeling is controlling DNA through histone acetylation. Histones are dynamic components of the transcriptional machinery that can be modified by post-translational modifications such as acetylation, methylation and phosphorylation [18], [19]. This landscape of modifications plays a dynamic role in chromatin structure, as they may influence histone-DNA interactions that regulate genetic activities [20]. In addition, it has been shown that acetylation of the chromatin is a crucial scaffold for histone methyl transferases to amplify the complex milieu of epigenetic markers found in the cell [21].

In particular, the acetylation of the ε-amino group of lysines residues on the histone tail by Histones acetyltranferases (HAT) is tightly correlated to gene transcription during development [22] and conditions such as cancer, inflammatory lung diseases and viral infections [23]. Conversely, histone deacetylases (HDACs) reverse the modifications made on histone tails and this correlates with a repressive state of the chromatin that is linked to terminal differentiation and cell cycle exit [24].

HDACs are highly conserved enzymes with homologues in yeast, humans, *Xenopus,* and zebrafish [25]. HDACs are classified based on their homology with yeast HDACs. Class I HDACs (1–3, and 8, homologous to yeast RPD3) are nuclear, expressed widely, and play an important role in cell proliferation and survival. Class II HDACs (4–7, and 9–10) shuttle between the nucleus and cytoplasm and have tissue-specific functions. Furthermore, HDAC activity has been shown to be important during multiple aspects of animal development including stem cell differentiation [26] and heart [27] and skull [28] morphogenesis.

Because HDACs are transcriptional repressors that lack DNA binding domains, their specificity is mediated by direct interaction with transcriptional repressors in large multi-protein complexes containing components such as NuRD, CoREST, or Sin3 proteins [29]. In particular, Class I HDAC complexes containing Sin3 also interacts with Mad proteins to act as a repressor of gene transcription [30]. Mad is a repressor of gene expression belonging to the basic-region-helix-loop-helix-leucine zipper (bHLH-Zip) family of transcription factors that binds to E-box sequences on the DNA [31]. Thus, Mad proteins are important modulators of HDAC action, targeting HDAC activity to specific regions on the chromatin.

As epigenetic regulation of DNA has been shown to be important for modulating stem cell states, such regulation may also play an important role in complex organ regeneration *in vivo.* Previous studies have identified methylation states and histone demethylases as regulatory components of regeneration in *Xenopus* tadpole limbs [32] and zebrafish fins [33]. However, the role of acetylation and histone acetylases is unknown. In order to determine whether modulation of histone acetylation can regulate regeneration, we took advantage of the tractable *Xenopus* tail model and used molecular and pharmacological tools to show that HDAC activity is required for regeneration. Inhibition of HDAC function blocks regeneration by altering the histone acetylation state and results in aberrant expression of the downstream genes involved in driving regenerative outgrowth. Furthermore, HDACs likely associate with the transcriptional repressor Mad3 to regulate histone acetylation.

## Results

### HDAC1 and Mad3 are expressed during tail regeneration

**Figure 1:**
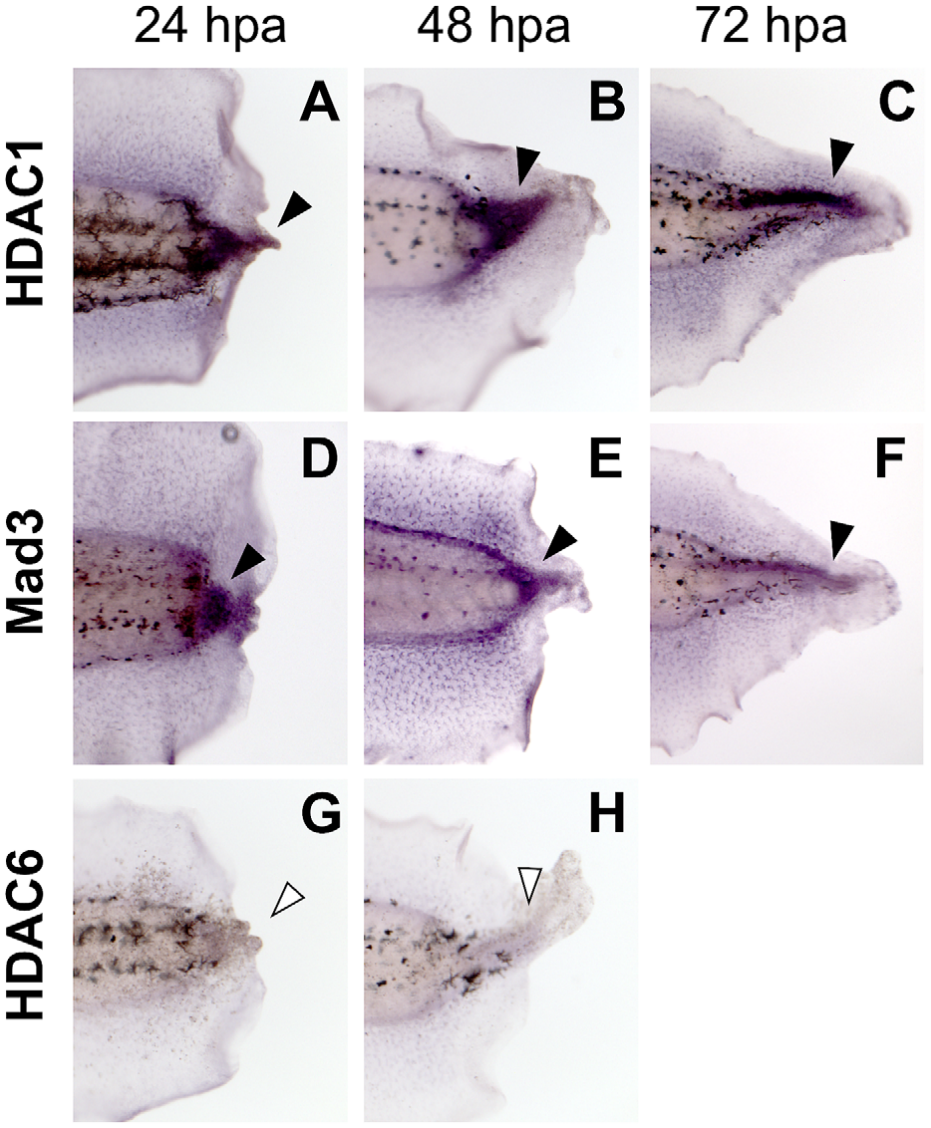
HDAC1 and Mad3 are Expressed During *Xenopus* Tail Regeneration. (**A–C**) RNA *in situ* hybridization to detect gene expression in tail regenerates at 24, 48 and 72 hpa for HDAC1, (**D–F**) Mad3, and (**G and H**) HDAC6. Probe targets are shown at the left. Black arrowheads indicate presence of RNA whereas open arrowheads indicate absence of expression. Anterior is to the left.

To determine whether HDACs play a role in regeneration, we first examined the endogenous expression patterns of Xenopus HDACs during regeneration. We identified full-length clones of *Xenopus* HDAC1 [34], and HDAC6 [35] using Xenbase [36]. We then examined their expression at several timepoints after tail amputation. RNA *in situ* hybridization with gene-specific probes showed that HDAC1 (also known as Rpd3) was strongly expressed at 24, 48, and 72 hpa (hours post amputation) in the mesenchymal cells of the regeneration bud (Fig. 1A–C, black arrowheads and Fig. S1) as compared to base levels of expression in flank (proximal tail) tissues. In contrast, HDAC6 expression was absent at both 24 and 48 hpa (Fig. 1G–H, open arrowheads). The difference in expression of HDAC1 and HDAC6 during regeneration suggests that, like their mammalian counterparts [22], HDAC function is likely not redundant and that subsets of HDACs play roles in different biological processes. While our expression data suggests specific HDACs as likely participants in regeneration, our studies do not rule out involvement of additional HDACs.

HDACs are known to associate with Mad3 to form a complex that regulates transcription; thus we also examined the expression pattern of Mad3 after tail amputation. Both HDAC1 and Mad3 RNAs were detected at low levels throughout the un-amputated tail, consistent with potential roles during primary tail development (Fig. S1). As expected, Mad3 RNA also becomes expressed in the regeneration bud at 24, 48, and 72 hpa (Fig. 1C, F). Together, our data indicate that HDAC1 and Mad3 are present in the correct spatiotemporal pattern to participate in appendage regeneration.

### HDAC function is required during early stages of tail regeneration

**Figure 2:**
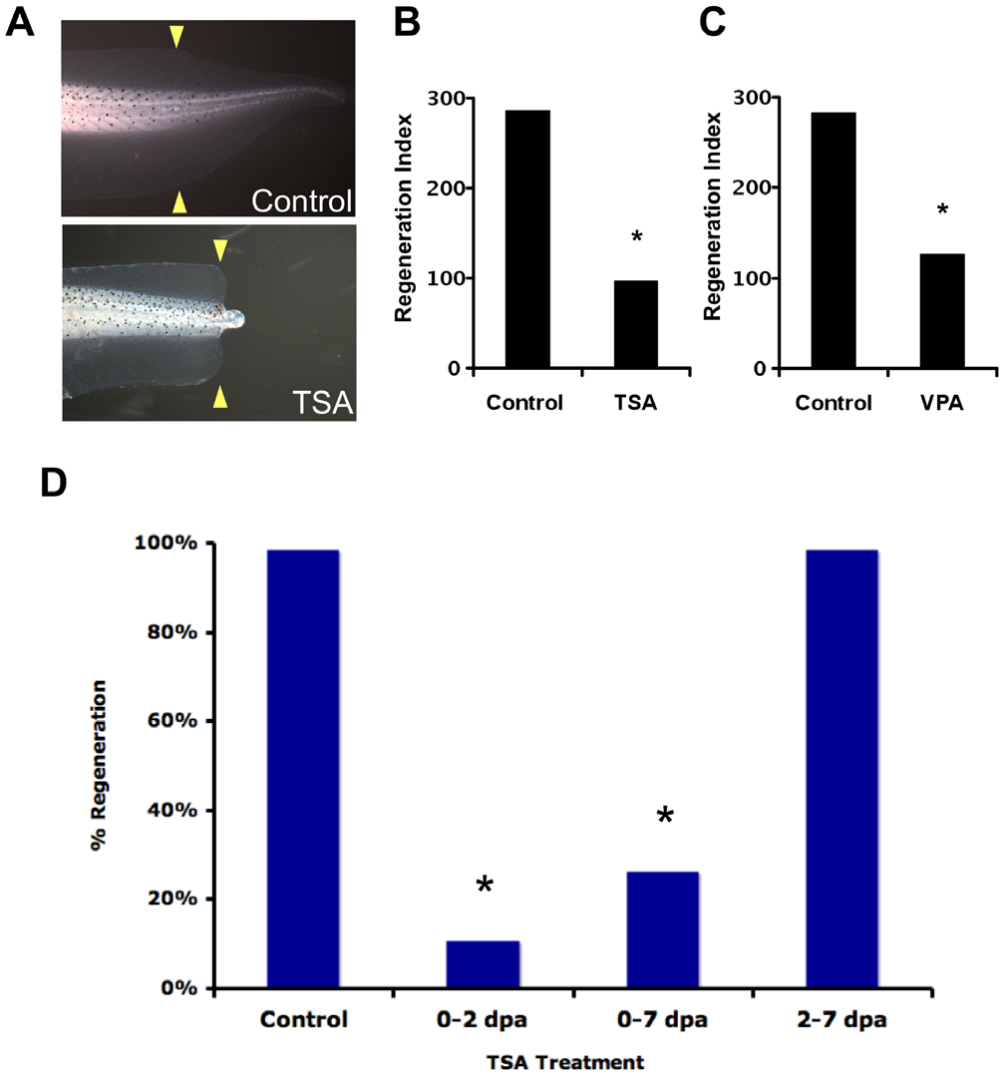
Pharmacological HDAC Blockade using TSA or VPA Inhibits Tail Regeneration. (**A**) After st. 40 tail amputation, tadpoles were assayed for tail regeneration at 7 days post amputation (dpa). Yellow arrowheads demarcate amputation site. (**B**) Control tadpoles (RI = 290, n = 69). 25 nM TSA treatment (RI = 109, n = 69), * denotes p<0.001. (**C**) Control tadpoles (RI = 283, n = 72). 500 µM Valproic Acid treatment (RI = 126, n = 53), * denotes p<0.001. (**D**) Temporal requirement for HDAC activity during regeneration. Percentage of regeneration shows total number of tail regenerates scored as full or good. TSA treatment as follows: Control/untreated (98.6%, RI =  290, n = 69), 0–2 dpa (10.8%, RI = 105, n = 65), 0–7 dpa (22.1%, RI = 118, n = 77), and 2–7 dpa (1.5%, RI = 272, n = 65). * denotes p<0.01 as compared to either Control or 2–7 dpa treatment.

To determine whether HDAC function is required for tail regeneration, we assessed the effect of pharmacologically ablating HDAC activity. To effectively block HDAC function, we used Trichostatin A (TSA), a well-known specific and potent chemical inhibitor of both Class I and Class II HDACs [37]. Tadpoles (whether control or amputated) that were treated with 25 nM TSA grew similarly to their untreated control siblings, and were indistinguishable with respect to developmental stage, axial proportions, gross organ morphology, and size (data not shown). After amputation at st. 40, tails of control larvae regenerated fully (Fig. 2A). In contrast, treatment with 25 nM TSA after tail amputation specifically inhibited regeneration (a decrease of 62% as determined by the Regeneration Index (RI), Fig. 2B). Similarly, treatment of st. 40 tadpoles after tail amputation with 500 µM of Valproic Acid (VPA), another well-characterized HDAC inhibitor [38], also significantly blocked regeneration (Fig. 2C). Together, these results demonstrated that HDAC activity is required for regeneration.

Our RNA expression data showed that HDAC1 is present during the events occurring right after tail amputation. Thus, we tested the hypothesis that HDAC activity is required during the early stages of regeneration by determining the temporal requirement for HDAC function. Tadpole tails were amputated and incubated for specific durations with TSA and assayed for their regenerative ability at 7 dpa (days post amputation) (Fig. 2D). Treatment through the entire length of the assay was sufficient to inhibit regeneration in 78% of tails (regenerates scored as weak or none) when compared to control siblings (1%) with no effects on overall development. Our RNA expression data showed that HDAC1 is expressed during the first two days of regeneration. Consistent with this observation, TSA treatment for the first 2 days after amputation caused 89% inhibition of tail regeneration. This result fully recapitulates the phenotype seen when the blocker was present for the entire duration of the assay. Further supporting an early role for HDACs, addition of TSA after 2 dpa had no effect on tail regeneration (1%), similar to control siblings. Together, our results demonstrate that HDAC activity is required specifically during the first 2 days of regeneration.

### Mad3 is required during regeneration

Class I HDACs are widely expressed transcriptional repressors that lack DNA binding domains. Thus their specificity is due to direct interaction with transcriptional repressors in large multi-protein complexes harboring NuRD, CoREST, or Sin3 proteins [29]. In particular, the HDAC-Sin3 complex also associates with the transcriptional repressor Mad proteins to regulate gene transcription [30].

Our previous work showed that Mad3 and the maternal HDAC functionally interact in the establishment of left-right asymmetry during early *Xenopus* development [39]. As Mad3 is expressed following tail amputation (Fig. 1D–F), it is a likely candidate for participation in the regenerative response. To further characterize the role of HDAC in regeneration, we looked to determine whether its partner Mad3 also participates in this process.

**Figure 3:**
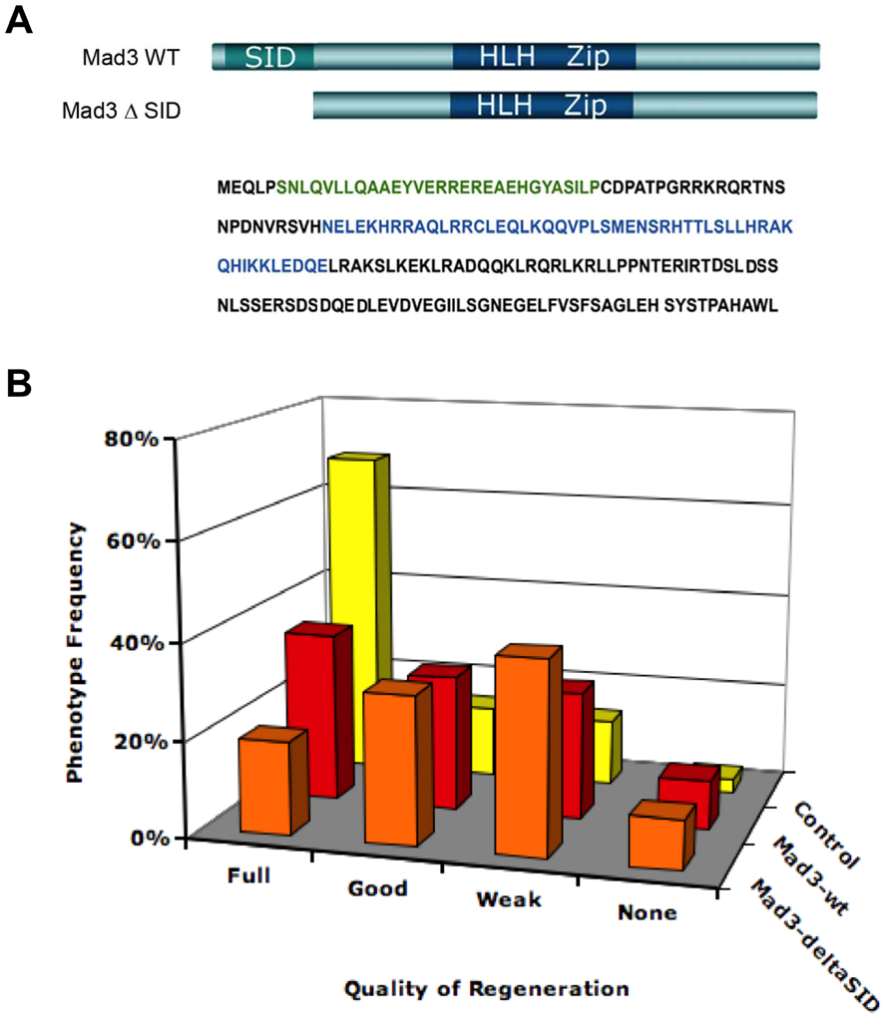
Over-Expression of Mad3 Inhibits Tail Regeneration. (**A**) Schematic showing the structure of Mad3 protein. Green indicates the Sin3-interacting domain (SID) important for the Mad3 interaction with HDACs. The DNA binding domain is represented in blue. Mad3 WT shows the full-length sequence and Mad3-ΔSID showing the construct that lacks the SID domain. (**B**) Mad3 WT, Mad3-ΔSID and lacZ mRNA were injected into early embryos at the 4 cell stage. Embryos were allowed to develop until st. 40, when tail amputation was performed. Graph showing effects of ectopic expression of Mad3 WT and Mad3-ΔSID on tail regeneration at 7 dpa. Control regenerates (RI = 250, n = 95). Wild-type Mad3 expression (RI = 188, n = 68, p<0.01). Mad3 with SID deletion expression (RI = 159, n = 88, p<0.01). p value denotes comparison to control. Comparison of the 2 ectopic expression experiments yielded p>0.05.

First, we characterized the potential role of Mad3 in regeneration. The over-expression of Mad3 has been shown to exert a dominant-negative effect on the HDAC1-Mad3 complex [39] and thus decreases its activity (likely via a titration mechanism). To determine whether Mad3 function affects regeneration, wild-type Mad3 RNA was injected into each blastomere of embryos at the 4-cell stage and expressed ubiquitously. As predicted, ectopic expression of Mad3 decreased tail regenerative ability by 25% when compared to control siblings (Fig. 3B), indicating that Mad3 participates in this process.

The ability of Mad3 to repress gene expression is dependent on its Sin3-Interacting Domain (SID), which enables Mad3 to interact with Sin3 co-factors. Sin3 in turn, recruits HDAC1 multi-protein complexes containing Mad, leading to transcriptional repression [40]. Indeed, the repressive activity is of Mad3 is dependent on the presence of HDAC and is fully blocked by HDAC inhibitors [30]. Because we showed that tail regeneration is sensitive to a HDAC blockade by TSA and VPA (Fig. 2B–C), we hypothesized that the requirement for Mad3 in regeneration is dependent upon HDAC function. To test this hypothesis, we generated a mutant Mad3 construct carrying a deletion of SID (Fig. 3A). This mutant Mad3 can not interact with Sin3-HDAC and should thus block HDAC-dependent functions during embryogenesis. This was indeed observed: ectopic expression of Mad3-ΔSID RNA reduced regenerative ability by 36% when compared to control siblings (Fig. 3B). This result shows that the function of Mad3 in regeneration requires SID and likely its interaction with HDAC via Sin3.

### Inhibition of HDAC Function Alters Regenerative Gene Expression

**Figure 4:**
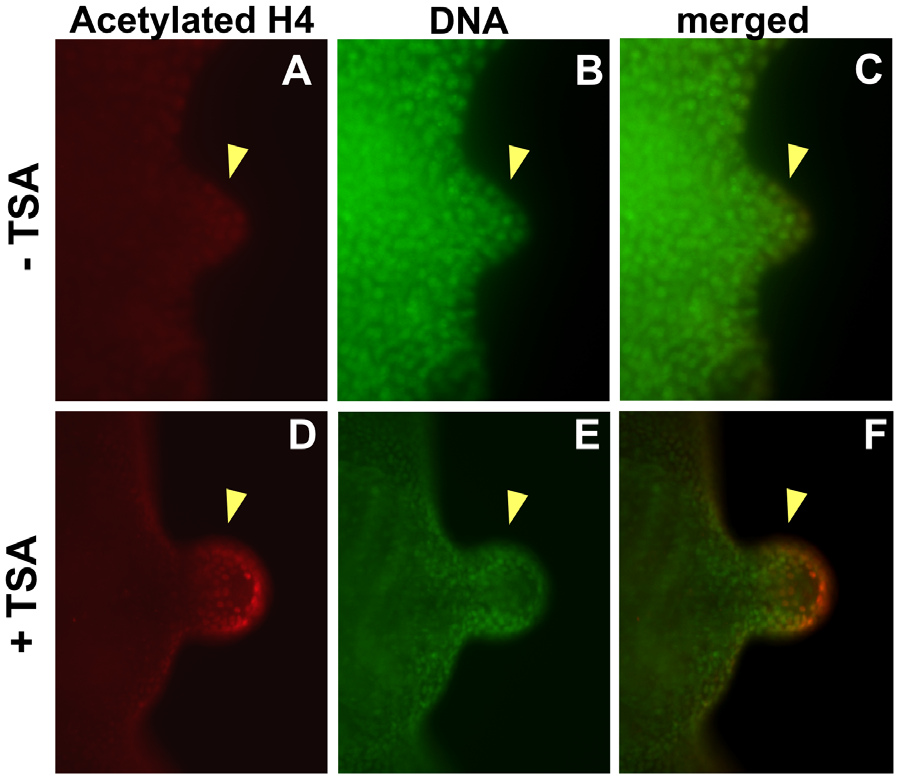
HDAC Inhibition Increases Histone Acetylation During Regeneration. The acetylation state of tail regenerates were examined at 24 hpa using an anti-acetylated Histone H4 antibody. Yellow arrowheads denote regeneration bud. Top row shows untreated controls. Bottom row shows tadpoles treated with 25 nM TSA after tail amputation. (**A, D**) Acetylated Histone H4. (**B, E**) Hoechst DNA stain. (**C**) merge of A and B. (**F**) merge of D and E.

Histone Deacetylases (HDAC) act to remove acetyl groups from the lysine amino acid on histones. To better understand the mechanism by which inhibition of HDAC activity blocks regeneration, we examined the effect of the HDAC inhibitor, TSA, on the acetylation state of the tail regenerate. Using an antibody that specifically detects acetylation on Histone H4, we observed a weak signal on control 24 hpa regeneration buds, consistent with our data that a low acetylation level of histone H4 is necessary for regeneration (Fig. 4A). In contrast, treatment with TSA immediately after tail amputation greatly increased the level of acetylation as seen by the strong acetylated Histone H4 signal in the regeneration bud (Fig. 4D compared to 4A). This result demonstrates that TSA acts to inhibit HDAC activity by altering the acetylation state of histones in the tail regeneration bud during regeneration.

The acetylation state of histones modulates genes expression. The removal of acetyl groups on histones by HDACs acts to repress transcription whereas the presence of acetyl groups enables transcription [41]. Our data indicated that HDAC inhibition abrogates tail regeneration by altering the acetylation state of histones. A likely reason for the regeneration defect is altered transcription of genes necessary for tail regeneration in regeneration bud. To assess the consequence of HDAC inhibition on key regenerative gene transcription, we examined the RNA expression pattern of Notch1 [13] and BMP2 [12], two genes that are required for promoting tail outgrowth after amputation. Individually activating either pathway promotes tail regeneration whereas inhibition prevents regeneration [13].

**Figure 5:**
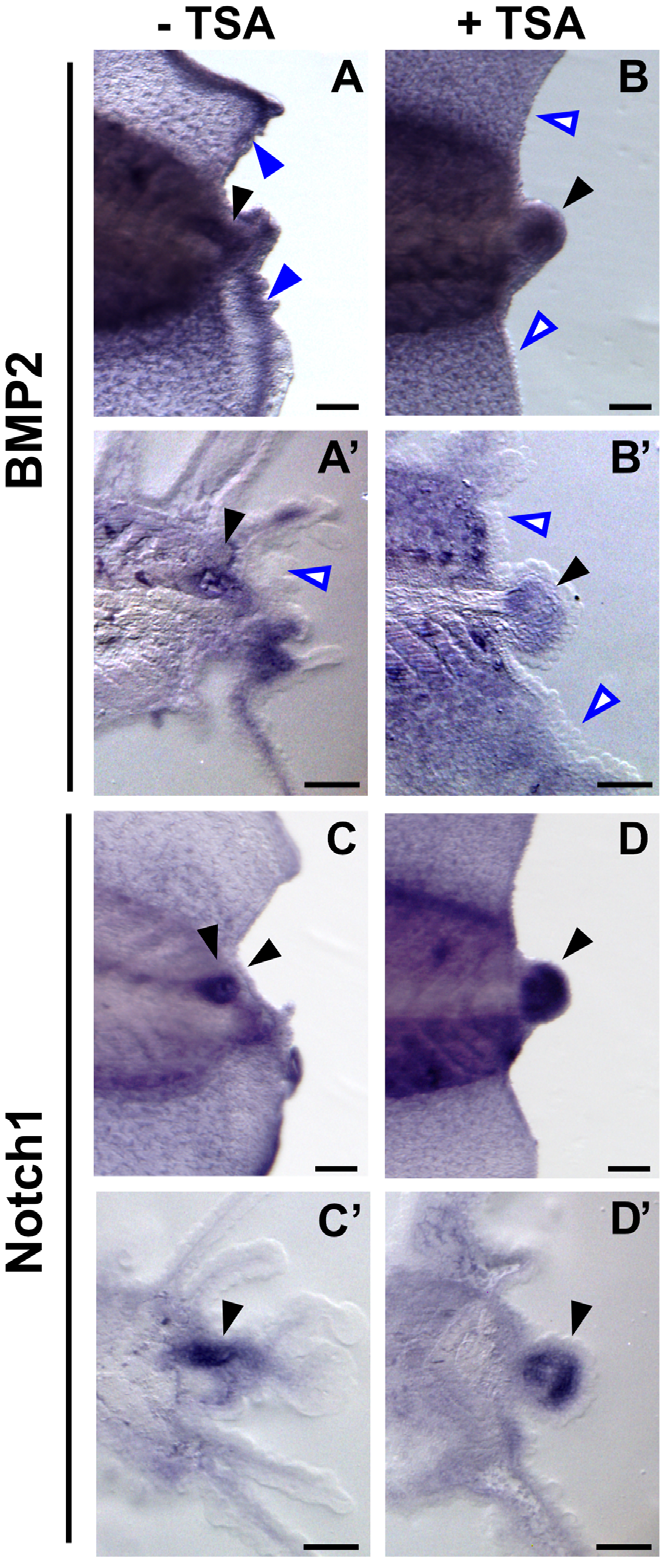
HDAC Inhibition Induces Mis-Expression of BMP2 and Notch1 During Regeneration. Wholemount *in situ* hybridization of st. 40 24 hpa regeneration buds. DIG-labeled probes specfic to *Xenopus* BMP2 and Notch1 were used to detect expression during regeneration without (**A, A', C, and C'**) or with (**B, B', D, and D'**) TSA treatment immediately after tail amputation. Wholemount views are shown in (**A, B, C, and D**) and sagittal sections through the regenerate tail are shown in (**A', B', C', and D'**). Anterior is to the left. Full arrowheads indicate gene expression. Open arrowheads show lack of expression. (**A, A', B, and B'**) BMP2. (**C, C', D, and D'**) Notch1. Scale bar = 100 µm.

At 24 hpa, *Xenopus* BMP2 is up-regulated in the regeneration bud (Fig. 5A and A', black arrowhead) and at the regenerating fin edge of the amputation site (Fig. 5A, blue arrowhead). Notch1 is expressed in the neural ampulla of the regeneration bud (Fig. 5C and C', black arrowhead). Addition of TSA after tail amputation resulted in expanded expression for both BMP2 and Notch1 in the regeneration bud (Fig. 5 B, B', D, and D', black arrowheads). Notably, expression of BMP2 at the regenerating tissue edge was lost (Fig. 5B and B', open blue arrowheads) suggesting that BMP2 expression during regeneration is regulated differentially by epigenetic control. Our data demonstrate that alteration in acetylation state causes mis-expression of regenerative gene programs.

The ectopic expression of genes that normally drive appendage regenerative outgrowth is counter-intuitive because HDAC inhibition blocks regeneration. However, our observations are consistent with other studies which have shown that TSA treatment increases BMP2 mRNA level in human osteoclasts [42] and also up-regulates Notch1 expression in cancer cells resulting in growth suppression [43]. Our observation that TSA treatment de-regulates normal regenerative gene expression further suggests that the proper control of gene expression pattern is an important requirement for regeneration.

## Discussion

Our results identify a novel role for HDAC activity during the early stages of *Xenopus* tail regeneration. We showed that a Class I HDAC, HDAC1, is highly expressed during endogenous regeneration but a Class II HDAC, HDAC6, is likely absent, although additional unidentified HDACs may also participate in regeneration.

The requirement for HDAC during regeneration is early, during the first two days following tail amputation. While the epigenetic control of DNA and its relationship to appendage regeneration is not well understood, previous studies have indicated that methylation states of gene enhancers may regulate regeneration. Work by Yakushiji and coworkers suggested that the epigenetic regulation of a gene expressed during regeneration, Sonic hedgehog (Shh), affects regenerative ability. They showed that the *Shh* enhancer, MFCS1, is hypomethylated during tadpole limb regeneration but is hypermethylated when the limb is unable to regenerate [32]. Moreover, Shh expression correlates strongly with the changes in DNA methylation state of the MFCS1 enhancer. Additionally, it has also been shown that a histone demeythlase is required for zebrafish fin regeneration [33]. Analogous to DNA methylation control, our study indicates that the establishment of a regenerative DNA acetylation state is important for enabling the correct spatial expression of genes that promote regeneration. A correct balance between DNA methylation and acetylation may be required to properly control regeneration.

Numerous studies support the hypothesis that HDACs can act as promoters of growth and proliferation [44]. For this reason, HDAC inhibitors have generated great interest and been pursued for their potential as anti-cancer therapies [45]. Our work is consistent with these previous studies in that treatment with TSA blocked tail regeneration. Surprisingly, we observed that inhibition of HDAC function caused aberrant expression of genes in pathways (BMP and Notch) that drive regenerative outgrowth (Fig. 4). This result was not unexpected since BMP2 mRNA has been demonstrated to increase in the presence of TSA treatment [42], and HDAC inhibitor treatment results in the up-regulation of Notch and suppressed cellular growth [43], [46]. Previous *Xenopus* work used constitutively-active forms of either the BMP receptor Alk3 or the intracellular active Notch domain (NICD) [13] to promote tail regeneration. Although the expression of BMP2 and Notch1 correlates with regenerative ability [8], it is not known whether the specific over-expression of BMP2 or Notch1 acts similarly.

Notably, our data show that HDAC function is critical for properly regulating the expression patterns of regenerative genes as an essential component of this process. Importantly, the direct regulation of Notch1 and BMP2 by histone acetylation is unlikely to account for the regenerative failure due to HDAC inhibition. It has been shown that HDAC inhibitor treatment in myeloma cells can modulate the mRNA levels of approximately 2% of expressed genes [47]. The identification of the affected genes during regeneration is of great interest, as they are likely to regulate the response to regeneration and coordinately act to re-grow the tail. It will be necessary to undertake global studies of acetylation states and corresponding microarray studies during the regenerative and non-regenerative states to identify these genes, as well as understand the interplay between genetic, epigenetic, and bioelectrical programs that are known to drive the regenerative response. A comprehensive understanding of this process will enable exciting new biomedical therapies for promoting regenerative repair of complex structures.

## Materials and Methods

### Tail regeneration assay

*Xenopus laevis* larvae were cultured via approved protocols (Institutional Animal Care and Use Committee, #M2008-08). Tails at stages (st.) 40–41 [48] were amputated at the midpoint between the anus and the tip. Tadpoles were separated into control or treated groups, to which 25 nM of Trichostatin A (TSA, Calbiochem) was added in 0.1X MMR at 22°C for 7 days and scored for tail regeneration. To quantify and compare regeneration in groups of tadpoles treated with or without TSA, a composite regeneration index (RI), ranging from 0 (no regeneration) to 300 (complete regeneration) was calculated as described previously [8]. Tail regenerates are scored into 4 phenotype categories (full, good, weak, none) (see Fig. S2). For example, a group of tails in which >80% were fully regenerated would have an RI ranging from 240 to 300; if full regeneration occurred in 10% of the animals within the group, the RI would range from 0 to 30.

### *Xenopus* embryo microinjection

For microinjections, capped, synthetic mRNAs [49] for *Xenopus* Mad3 and Mad3ΔSID were generated using the mMessage mMachine kit (Ambion). Embryos at the 4-cell stage were transferred to 0.1X MMR containing 3% Ficoll and synthetic mRNA was injected into each of the 4 blastomeres along with the lineage tracer rhodamine-labeled dextran (Invitrogen). At 30 minutes post-injection, embryos were transferred to plain 0.1X MMR and cultured at 18°C until they reached st. 40–41.

### In situ hybridization and immunohistochemistry

Sequence data for this paper were retrieved from Xenbase, University of Calgary, Alberta T2N 1N4, Canada; URL: http://www.xenbase.org/. Wholemount RNA *in situ* hybridization was performed according to standard protocols [50] with probes to *Xenopus* Mad3 (Open Biosystems clone ID: 4175511), HDAC1/Rpd3 (Open Biosystems clone ID:4683530), HDAC6 (Open Biosystems clone ID:5542663), Notch1 [51], and BMP2 [52]. Embedding of tails were performed using Polysciences JB-4 Embedding Kit according to the manufacturer's protocol. A Leica Microtome (VT1000S) was used to obtain 50 µm sections. *Xenopus* embryos were fixed overnight in MEMFA buffer [53], permeabilized in PBS and 0.1% Triton X-100 for 30 min, and processed for immunohistochemistry using fluorescent secondary detection (Invitrogen). Anti-acetyl Histone H4 antibody was purchased from Millipore.

### Statistical analysis

To compare tail regeneration experiments, raw data from scoring was used. Comparison of two treatments was analyzed with Mann–Whitney *U* test for ordinal data with tied ranks, using normal approximation for large sample sizes. Multiple treatments were compared using a Kruskal–Wallis test, with Dunn's Q corrected for tied ranks.

## Supporting Information

Figure S1 **Expression of HDAC1 and Mad3 in** ***Xenopus*** **Tail.** RNA *in situ* hybridization to detect gene expression in st. 40 uncut and amputated tail for (**A–B**) HDAC1, (**C–D**) Mad3, and (**E–F**) β-gal. Probe targets are shown to the left of the panels.
(TIF)

Figure S2 **Tail Regeneration Assay.** Individual animals for each specific treatment were scored as follows: Full: complete regeneration. Good: robust regeneration with minor defects (missing fin, curved axis). Weak: poor regeneration (hypomorphic/defective regenerates). None: no regeneration. Shown are representative examples of each regenerate class.
(TIF)
