# Supplementary figures and images for: HDAC Activity Is Required during Xenopus Tail Regeneration
Source: PLoS One. 2011 Oct 14;6(10):e26382. doi: 10.1371/journal.pone.0026382 (PMC3194833; doi:10.1371/journal.pone.0026382)

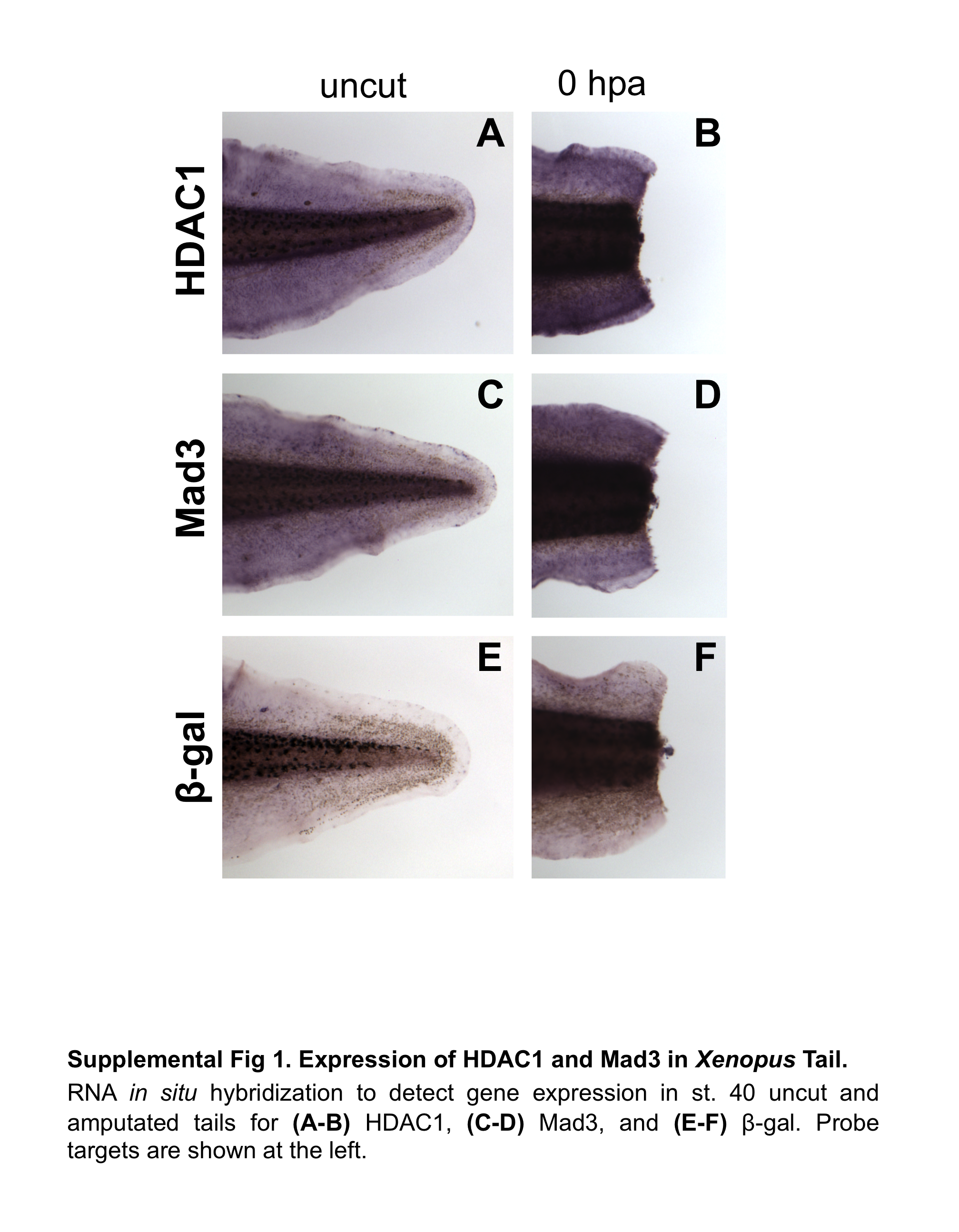

Supplement: Figure S1 — Expression of HDAC1 and Mad3 in Xenopus Tail. RNA in situ hybridization to detect gene expression in st. 40 uncut and amputated tail for (A–B) HDAC1, (C–D) Mad3, and (E–F) β-gal. Probe targets are shown to the left of the panels. (TIF) [file pone.0026382.s001.tif]

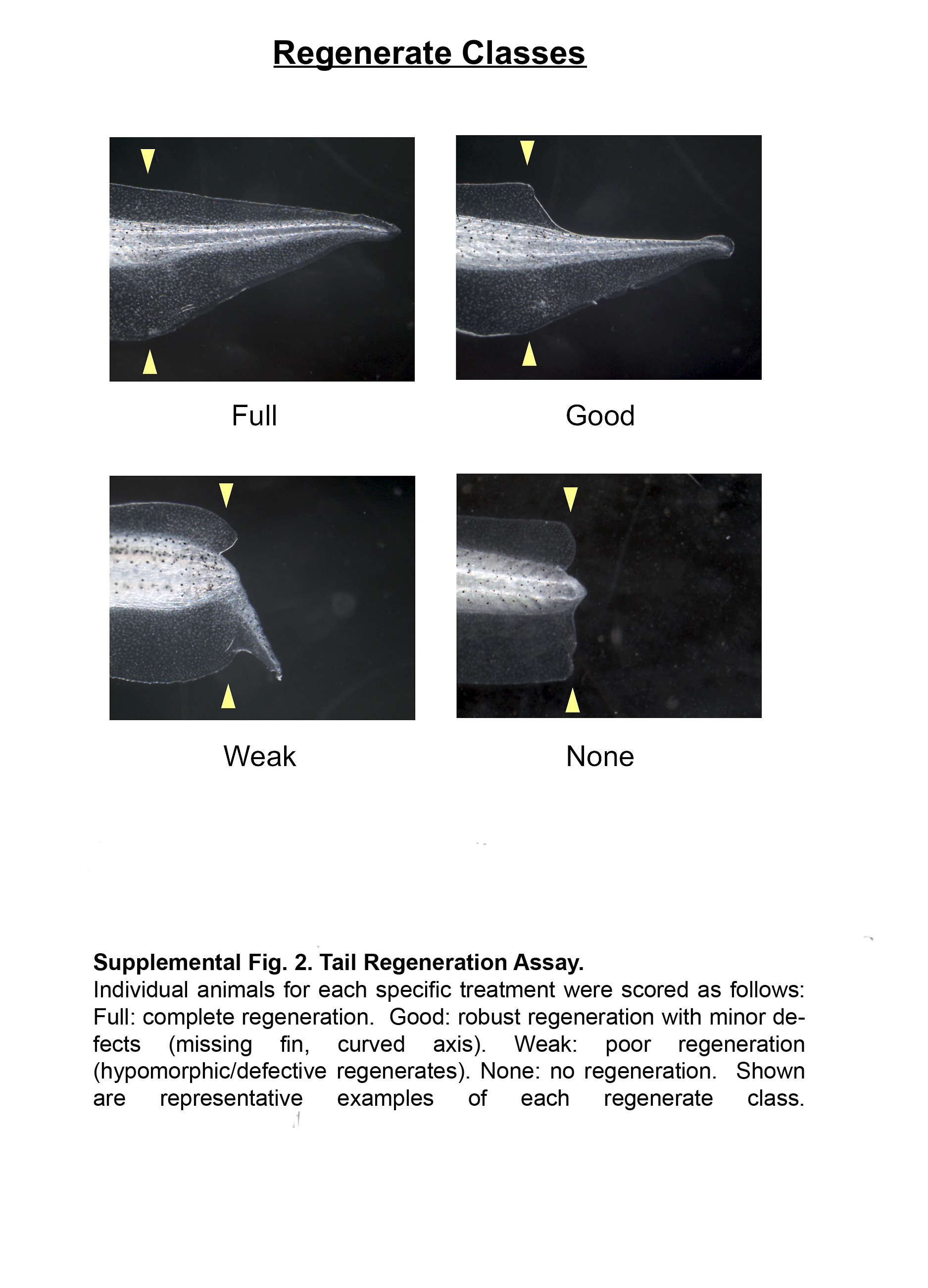

Supplement: Figure S2 — Tail Regeneration Assay. Individual animals for each specific treatment were scored as follows: Full: complete regeneration. Good: robust regeneration with minor defects (missing fin, curved axis). Weak: poor regeneration (hypomorphic/defective regenerates). None: no regeneration. Shown are representative examples of each regenerate class. (TIF) [file pone.0026382.s002.tif]
